# Letter to the editor: Pending challenges in passenger contact tracing in air transport – a German perspective

**DOI:** 10.2807/1560-7917.ES.2019.24.32.1900498

**Published:** 2019-08-08

**Authors:** Juliane Seidel, Dorothea Matysiak-Klose, Matthias Jeglitza, , Nadine Litzba

**Affiliations:** 1 Robert Koch Institute, Berlin, Germany; 2 Federal Ministry of Transport and Digital Infrastructure of Germany, Berlin, Germany

**Keywords:** air travel, passenger contact tracing, legislation, International Health Regulations, airline, public health

**To the editor:** Inspired by the recently published article ‘Contact tracing following measles exposure on three international flights, Germany, 2017’ [1], which exemplarily describes the challenges encountered in contact tracing of air passengers after measles exposure, we want to add a broader, Germany-wide perspective and highlight the need for long-term international solutions.

Generally, the main aim of performing contact tracing is to prevent further spread of infectious diseases in populations by conducting timely public health measures (e.g. measles post-exposure prophylaxis needs to be provided within 3 days (for vaccination) or 6 days (for immunoglobulins) after exposure). Contact tracing is a resource-intensive process initiated by a public health authority (PHA) after a careful decision-making process. Before contacting the affected passengers, the PHA needs to contact the airline responsible for the respective flight in order to obtain the contact information of passengers and crew members. The PHA must also inform all other German federal-state and local PHAs concerned so that they can contact the affected passengers residing in their respective areas of responsibility. In cases of non-German passengers, the respective national focal point has to be contacted as well.

Despite the existing German [2,3] and international legal frameworks [4-6], the German PHAs face obstacles at all levels. Airlines often question the lawfulness of passenger data disclosure and are frequently uncertain of their legal responsibilities, which may result from the dispersion and variety (national, European Union (EU) and international level, as well as different areas of operation) of the aforementioned laws.

In most cases it is difficult for PHAs to find sustainable and reliable contacts with sufficient expertise at the interface between aviation and public health protection. While this may still be possible with large airlines and national carriers, it can be extremely difficult with uncommon third-country operators or some low-cost providers. In addition, airlines do not necessarily have a valid data record of their passengers. Depending on the business model and the booking systems used, sometimes hardly any reliable passenger data are available for PHAs; this is even more likely if passengers’ flights were booked as part of a travel group. Consequently, PHAs cannot fulfil their legal obligation to implement public health measures. Additionally, the existing European passenger name record (PNR) is neither designed nor available as a source of information for public health purposes [7].

These challenges result in time-consuming processes that tie up resources that are urgently needed elsewhere and lead to delayed or no longer effective public health measures. Therefore, these issues are recurrently brought up by federal, state and local PHAs in different committees.

In our view, at least the following steps are needed to effectively enforce public health protection in the civil aviation sector: (i) establish a general understanding that public health issues are just as important as other public concerns; (ii) develop a common understanding among all stakeholders of the legal basis and importance of the issue at all technical levels, e.g. a memorandum of understanding between PHAs and airlines; (iii) agree upon a standard for booking data that is sufficient to clearly identify and contact passengers for defined legal purposes and (iv) ensure that these data are accessible to PHAs 24/7 via a single-window system, which enables all involved stakeholders to use one system to provide and retrieve information.

These suggestions may contribute to reducing the amount of time and resources required of the involved stakeholders and, perhaps more importantly, to preventing further spread of infectious diseases.
